# Anemia and Microvascular Complications in Patients With Type 2 Diabetes Mellitus

**DOI:** 10.5812/numonthly.19976

**Published:** 2014-07-05

**Authors:** Mahboobeh Sadat Hosseini, Zohreh Rostami, Alireza Saadat, Sayyed Mehdi Saadatmand, Effat Naeimi

**Affiliations:** 1 Nephrology and Urology Research Center, Baqiyatallah University of Medical Sciences, Tehran, IR Iran; 2 Department of Hematology, School of Medicine, Baqiyatallah University of Medical Sciences, Tehran, IR Iran; 3 Students Center for Medical Research, Baqiyatallah University of Medical Sciences, Tehran, IR Iran; 4 Endocrinology and Metabolism Department, Baqiyatallah University of Medical Sciences, Tehran, IR Iran

**Keywords:** Anemia, Diabetes Mellitus, Microvascular Complications

## Abstract

**Background:**

Although chronic kidney disease-induced anemia is more prevalent in patients with diabetes mellitus (DM), anemia is a common finding prior to manifestation of kidney disease. In presence of some risk factors at the time of diagnosing DM, microvascular complications must be considered. The effect of anemia as a risk factor on progression of DM complications is still unclear.

**Objectives:**

The aim of the study was to determine the prevalence of anemia and its association with microvascular complications in patients with type 2 DM.

**Patients and Methods:**

This cross-sectional study was performed in the outpatient endocrinology clinic at Baqiyatallah University of Medical Sciences Hospital, Tehran, Iran. Study was done from February 2011 to February 2012. Patients with type 2 DM without any obvious symptom or sign of anemia were included in study.

**Results:**

A total of 93 patients (30.4%) had anemia including 46 (15.1%) with normochromic normocytic, 44 (14.4%) with hyperchromic microcytic, and 3 (1%) with hyperchromic macrocytic anemias. There was a positive correlation between duration of DM and anemia. Microvascular complications were more frequent with normocytic or microcytic anemias. Glomerular filtration rate (GFR) was higher in patients without anemia; moreover, nephropathy was less frequent among them. Among patients with anemia, 43% had GFR of more than 90 mL/min and 19.4% had normoalbuminuria. Neuropathy, nephropathy, and retinopathy had strong association with anemia (odds ratio of 1.99, 1.7, and 1.5, respectively).

**Conclusions:**

Anemia is a common complication of DM and is associated with duration of disease and microvascular complications.

## 1. Background

Economic development, changes in lifestyle, and improvement in life expectancy have resulted to increasing percentage of diabetes mellitus (DM) in general population (1). if the current trends continue in such a way, one out of three adults in the United States might have DM by 2050 (2). Owing to high prevalence of microvascular and macrovascular complications, type 2 DM has a signiﬁcant association with comorbidities that have developed before diagnosing DM (3). In different studies, prevalence of microvascular complications among newly diagnosed patients with DM ranges from 5% to 35% (3-6); however, microvascular complications in the presence of some risk factors at the time of diagnosing DM have been considered in few studies (3). Overall, the level of albuminuria, serum albumin, serum creatinine, and hemoglobin (Hb) are the most important risk factors associated with microvascular complications (7). Although chronic kidney disease-induced anemia is more prevalent in this population, anemia is a common finding prior to kidney disease development (8, 9). Almost 7% of outpatients with DM have a Hb level of less than 11 g/dL (10). In another study, 20% of ambulatory patients with type 1 or type 2 DM presented with anemia (9). In a study in Iran, prevalence of anemia was estimated at 10% in patients with type 2 DM (11). Although anemia could be indicative of diabetic kidney disease, reduced Hb levels, even within the normal range, can identify diabetic patients with an increased risk of microvascular complications, morbidity, and mortality. The outcome of heart failure and hypoxia-induced organ damage in patients with DM can also be affected by severe anemia (9). Anemia is generally associated with lower quality of life. In patients with renal impairment, it significantly contributes to morbidity and symptoms such as lack of energy, breathlessness, dizziness, poor appetite, reduced cognitive function, and decreased exercise tolerance (9). The effect of anemia on progression of diabetic complications is still unclear. Correction of anemia in patients with DM must be studied in further clinical trials.

## 2. Objectives

The present study aimed to report the prevalence rate of various microvascular complications of type 2 DM and anemia-related complications.

## 3. Patients and Methods

### 3.1. Patients

This cross-sectional study was performed in the outpatient endocrinology clinic of Baqiyatallah University of Medical Sciences Hospital. We recruited patients with known type 2 DM from the waiting list of endocrinology clinic or from those suspected for DM at the first visit. Patient recruitment was according to American Diabetes Association (ADA) criteria (12). General practitioner researchers invited patients to participate in this study. From 372 potential eligible patients, seven refused to participate, and 21 were excluded. From the remaining 344 eligible patients, 305 were recruited according to a predetermined sample size. Patients’ demographic characteristics, medical history, results of clinical examinations, and paraclinical studies were investigated. A board-certified specialist in endocrinology assessed participants with eligibility criteria and conducted further studies if needed. Patients with obvious cause of anemia such as thalassemia, end-stage renal disease, chronic inflammatory disease, rheumatoid arthritis, infection, pancreatitis, glucose-6-phosphate dehydrogenase deficiency, hemolysis, and acute or chronic blood loss as well as heavy smokers were excluded from the study.

### 3.2. Examination and Laboratory Studies

For each patient height (in standing position without shoes, using tape meter with shoulder in normal state), weight (minimally dressed without shoes, using digital scales recorded) and body mass index (BMI; weight in kg divided by square height in m^2^) were measured. Neurologic examinations were done by Michigan Neuropathy Screening Instrument. Score above two was indicative of neuropathy presence (12). To assess retinopathy, all patients were referred to an ophthalmologist and the degree of eye involvement including proliferative and nonproliferative diabetic retinopathies was determined. Ophthalmologic examination was done using indirect ophthalmoscopy with dilated pupils. To determine the prevalence of true albuminuria, we tried to eliminate other factors inducing transient albuminuria such as urinary tract infection, sever hypertension; sever hyperglycemia, exercise, and acute fever. If these factors could not be eliminated, patients were excluded. In addition, 24-hour urine volume, creatinine (Cr), albumin, fasting blood sugar (FBS), glycosylated Hb (HbA1C), complete blood count (CBC), Cr, and C reactive protein (CRP) were measured. Albumin levels more than 30 mg in 24 hours was considered as albuminuria (12). Creatinine clearance (Cl_Cr_) in men was calculated using Cockcroft-Gault equation: 

Cl_Cr_ (mL/min) = [(140 - age) × weight (kg)]/[serum creatinine (μmol/L) × 72]

For women, the results were multiplied by the coefficient of 0.85. Anemia was considered as Hb < 13 g/dL in men and < 12 g/dL in women. Mean corpuscular volume (MCV) of more than 100 fL and less than 80 fL were considered as macrocytic and microcytic anemia. Normocytic anemia was defined as MCV value between 80 and 100 fL. Results of the laboratory tests that were measured outside the hospital were excluded. CBC differential normal values, Cr, FBS, and albuminuria were measured by using cell counter device (Biosystem Company Kits), glucose oxidase, Jaffe, and photometer methods, respectively.

### 3.3. Ethical Considerations

The study was conducted in accordance with the Declaration of Helsinki and was approved by the Ethics Committee of Baqiyatallah University of Medical Sciences. The purpose of the study was explained to all participants. Patients were informed that they were free to withdraw from the study at any time. A nurse accompanied patients for laboratory sampling and if needed, provided verbal information for participants. A written informed consent was given to all patients at the first study visits and they did not pay for diagnostic tests. They were referred to other departments for appropriate treatment when needed.

### 3.4. Sample Size and Statistical Analyses

Sample size calculations were based on this formula: 

N = 4 × (pq/ω^2^) z_1-/2_^2^,

Where p was the anticipation of anemia prevalence; q = 1 - p; ω was the planned width of 95% confidence interval (CI) for estimation of prevalence; α = 0.05; and z_0.975_ = 1.9600. 

For the anticipated prevalence of 23%, we included 305 participants to provide the planned width of 95% CI (ω = 0.1).

### 3.5. Statistical Analysis

Data were presented as mean and standard deviation for continuous variables and as numbers and percent for categorical variables. The data were tested for normality with the help of histograms, comparison of means and medians, skewness, skewness/standard error, and the Kolmogorov-Smirnov test with Lilliefors significance correction. In case of normal distribution the independent-samples t-test was used; otherwise, the Mann-Whitney U test was performed. Chi squared test was used for analysis of categorical data. Correlations between variables were assessed as odds ratios and 95% CI by logistic regression models. Data were analyzed with SPSS for Windows (version 15, SPSS, Inc., Chicago, IL, USA). A p value of less than 0.05 was considered as statistically significant, and the power of statistical tests was set at 80%. We did not include patients with missing data in the statistical analysis.

## 4. Results

From a total of 305 participants with mean DM duration of 8.2 ± 7.1 years and mean age of 53.9 ± 1 years, 47.2% were males. Table 1 summarized the characteristics of the patients according to their sex. Overall, 76% of patients was on oral hypoglycemic agents and the remained patients received insulin or combined insulin and oral agent treatment. More than two-thirds of patients presented with at least one of the DM-associated microvascular complication. The prevalence of retinopathy, neuropathy, and nephropathy was 35.7%, 44.6%, and 77%, respectively. Anemia was detected in 93 patients (30.4%) with normochromic normocytic in 46 patients (15.1%), hypochromic microcytic in 44 (14.4%), and hyperchromic macrocytic anemia in 3 (1%). Table 2 shows microvascular complications regarding anemia in study population. Nephropathy was more prevalent than other complications in patients with anemia (80.6% vs. 34.4%); moreover, those with anemia had longer DM duration as well as lower GFR. The association between nephropathy and anemia is described in details in Table 3. Correlation between type of anemia and microvascular complications is showed in Table 4. Among patients with anemia, 43% had GFR > 90 mL/min and 19.4% had normoalbuminuria.

Logistic regression analysis showed that anemia might be a predictor of microvascular complications (Table 5). In this study, medication used for management of DM including oral hypoglycemic medication, Statin, and aspirin had no significant effect on complications (P value of 0.129, 0.9, and 0.18, respectively). In comparison to other patients, those with anemia frequently used insulin (P < 0.0001), angiotensin converting enzyme inhibitors (P = 0.028), and angiotensin receptor blockers (P = 0.029).

**Table 1.** Patient Characteristics With Respect to Sex ^a,b,c^

	Men	Women	Total
**Gender**	144 (47.2)	161 (52.8)	305
**Age, y**	55 ± 10.9	52.8 ± 9.1	53.9 ± 1.0
**Duration of Diabetes, y**	7.7 ± 6.9	8.6 ± 7.3	8.2 ± 7.1
**BMI, kg/m** ^**2**^	26.48 ± 3.3	28 ± 4.6	27.5 ± 4.3
**Hb, g/dL**	14 ± 1.7	12.7 ± 1.4)	13.4 ± 1.7
**Nephropathy**	112 (77.8)	123 (76.4)	235 (77.0)
**Neuropathy**	63 (43.8)	73 (45.3)	136 (44.6)
**Retinopathy**	52 (36.1)	57 (35.4)	109 (35.7)
**GFR, mL/min**	81.3 ± 27.8	79.52 ± 29.4	80.6 ± 28.6
**Oral Hypoglycemic Agents**	112 (48.3)	120 (51.7)	232 (76.0)
**Insulin**	29 (55.7)	23 (44.2)	52 (17.0)
**Insulin Plus Oral Hypoglycemic Agents**	9 (6.3)	10 (6.2)	19 (6.2)
**ACEI**	39 (27.1)	36 (22.4)	75 (24.6)
**ARB**	30 (20.8)	30 (18.6)	60 (19.7)
**Statin**	60 (41.7)	67 (41.6)	127 (41.6)
**Aspirin**	78 (54.2)	80 (49.7)	158 (51.9)

^a^ Abbreviations: BMI, body mass index; Hb, hemoglobin; GFR, glomerular filtration rate; ACEI, angiotensin converting enzyme inhibitor; and ARB, angiotensin receptor blockers.

^b^ Data are presented as mean ± SD or No. (%).

^c^ Two patients did not receive any drug.

**Table 2.** Patients’ Characteristics With Respect to Anemia^a,b^

	No anemia, n = 212 (69.5%)	Anemia, n = 93 (30.4%)	Total n = 305
**Gender**			
Male	104 (49.1)	40 (43.0)	144 (47.2)
Female	108 (50.9)	53 (57.0)	161 (52.8)
**Age, y**	52.9 ± 9.9	56.1 ± 10.2	53.9 ± 1.0
**Duration of Diabetes, y**	6.8 ± 6.5^c^	11.4 (7.5)	8.2 ± 7.1
**BMI, kg/m** ^**2**^	27.2 ± 4.5	28.2 ± 3.8)	27.5 ± 4.3
**Hb, g/dL**	14.2 ± 1.2	11.4 ± 0.9	13.4 ± 1.7
**Nephropathy**	74 (34.4)^c^	75 (80.6)	235 (77.0)
**Neuropathy**	75 (35.4)^c^	61 (65.6)	136 (44.6)
**Retinopathy**	61 (28.8)^c^	48 (51.6)	109 (35.7)
**GFR, mL/min**	84.5 ± 27.3^c^	71 ± 29.5	80.6 ± 28.6
**Oral Hypoglycemic Agents**	173 (81.6)	59 (63.4)	232 (76.0)
**Insulin **	26 (12.2)^c^	26 (27.9)	52 (17.0)
**Insulin Plus Oral Hypoglycemic Agents **	8 (3.8)^c^	11 (11.8)	19 (6.2)
**ACEI**	45 (21.0)^c^	30 (45.0)	75 (24.6)
**ARB**	35 (16.5)^c^	25 (26.9)	60 (19.7)
**Statin**	89 (42.0)	38 (40.9)	127 (41.6)
**Aspirin**	114 (53.8)	44 (47.3)	158 (51.8)

^a^Abbreviations: BMI, body mass index; Hb, hemoglobin; GFR, glomerular filtration rate; ACEI, angiotensin converting enzyme inhibitor; and ARB, angiotensin receptor blockers.

^b^Data are presented as mean ± SD or No. (%).

^c^ P value < 0.05.

**Table 3.** Correlation Between Anemia and Nephropathy^a,b^

	Study Groups			
	Anemia	No anemia	Total	P value
**GFR, mL/min**	71 ± 29.5	84.5 ± 27.3		0.0001
> 90	40 (43.0)	116 (54.7)	156 (51.1)	-
60-89	18 (19.3)	59 (27.8)	77 (25.2)	-
30-59	27 (29.0)	34 (16.0)	61 (20.0)	-
< 30	8 (8.6)	3 (1.4)	11 (3.6)	-
**24-Hour Urine Albumin, mg/24h**	290 ± 50	270 ± 60		0.0001
< 30	18 (19.4)	138 (65.1)	156 (51.0)	-
30-299	42 (45.2)	52 (24.1)	94 (30.4)	-
> 300	33 (35.5)	22 (10.4)	55 (18.1)	-

^a^Abbreviation: GFR, glomerular filtration rate.

^b^Data are presented as mean ± SD and No. (%).

**Table 4.** Correlation Between Type of Anemia and Microvascular Complications^a^

Type of Anemia	Nephropathy		Neuropathy		Retinopathy	
	Yes	No	Yes	No	Yes	No
**Macrocytic**	2 (2.6)	1 (5.5)	2 (3.3)	1 (3.1)	2 (4.2)	10 (22.2)
**Normocytic**	45 (60.0)^b^	7 (38.8)	26 (42.6)	23 (71.8)	23 (47.9)^b^	18 (40.0)
**Microcytic**	28 (37.3)	10 (55.5)	33 (54.0)^b^	8 (25.0)	23 (47.9)^b^	17 (37.7)
**Total**	75 (80.6)^b^	18 (19.4)	61 (65.6)^b^	32 (34.4)	48 (51.6)^b^	45 (48.4)

^b^Data are presented as No. (%).

^a^P value < 0.05.

**Table 5.** Effects of Anemia on the Probability of Microvascular Complications^a^

Risk Factor	Adjusted Odds Ratio (95% CI)
**Age**	0.99 (0.97-1.04)
**The Duration of Diabetes**	0.96 (0.92-1.00)
**ACEI or ARB use**	1.23 (0.64-2.28)
**Insulin Use **	1.38 (0.69-2.75)
**Nephropathy**	1.7 (1.20-2.00)
**Neuropathy**	1.99 (1.40-3.60)
**Retinopathy**	1.5 (1.20-1.90)

^a^Abbreviations: ACEI, angiotensin converting enzyme inhibitor; and ARB, angiotensin receptor blockers.

## 5. Discussion

We found that about one-third of patients with type 2 DM (28% of males and 33% of females) had anemia with normochromic normocytic anemia being the most frequent type. Microvascular complications were more common in patients with normocytic or microcytic anemia. Nephropathy was the most common complication. Patients with anemia had lower GFR. Our data highlighted the effect of anemia. In patients with diabetic nephropathy, anemia might be a significant cause of referring to nephrology services. Odds ratio for nephropathy, neuropathy, and retinopathy, in patients with anemia was significantly higher in comparison with patients without anemia.

Normocytic anemia was the most prevalent type of anemia in patients with nephropathy. After adjustment for some risk factors, relative risk of microvascular complications in patients with anemia was 1.5-time to two-time more prevalent than in patients without anemia. Previous studies showed anemia as a common morbidity in diabetic kidney disease and introduced anemia as an independent predictor of progression to end-stage renal disease (13, 14). Even reduced Hb in normal or lower limit of normal range contributes to incising risk of microvascular complications (7). Although the mechanism by which anemia might affect progression of kidney disease is currently unknown, altered oxygen delivery to interstitial structures of the kidney and changes in cardiac function could potentially be important (14). Tubulointerstitial damage would be followed by destruction of interstitial fibroblasts, tubular atrophy, and glomerulosclerosis. Systemic inflammation, functional hematinic deficiencies, erythropoietin resistance, and reduced red cell survival also lead into anemia in the setting of impaired renal compensation (8). Due to different eligibility criteria and various definitions of anemia, different values of prevalence have been reported in the literature (11, 15-18). Similar to our study, other studies have shown that anemia can increase the risk of microvascular complications (9, 18-21). In the present study, 43% of patients with anemia had GFR of more than 90 mL/min and 19.4% had normoalbuminuria. These data were consistent with the fact that anemia is a multifactorial condition and could be detected before demonstrable decline in renal function (17, 22-24).

Several mechanisms have been proposed for the association between DM and anemia (25). Autonomic neuropathy can decrease sympathetic stimulation of erythropoietin production through renal denervation. Moreover, the effect of DM on tissues responsible for synthesis of erythropoietin reduces kidney response to hypoxia. Decrease in androgenic hormones level in DM attenuates stem cells in bone marrow and reduces erythropoietin synthesis in kidney. According to one study, reticulocytes show resistance in their response to increased levels of erythropoietin (26). Diabetic patients without nephropathy have an appropriate erythropoietin response to hypoxia. An important cause of resistance to erythropoietin is the inflammation accompanied by the rise of cytokines and consequent suppression of erythrocyte stem cell proliferation. The study suggested that before development of nephropathy, overt inflammation associated with diabetes may culminate erythropoietin suboptimal response. Mean GFR in our patients with anemia was 71 mL/min. Anemia becomes increasingly common as GFR declines below 60 mL/min/1.73 m^2^ (27-29). As mentioned before, anemia may ensue destruction of peritubular fibroblasts and decrease in erythropoietin level even before detectable reduction in GFR (8, 25). Like other studies, our research showed that albuminuria and decrease in GFR were the major predictors of anemia. In our sample, 80.7% of patients with anemia had more than 30 mg/24h albuminuria and 56.9% of patients with anemia had GFR of less than 90 mL/min (9, 11, 17). Retinopathy and neuropathy were also more common in patients with anemia. In addition to renal failure, some factor such as inflammations, nutritional insufficiency, malnutrition, anemia due to chronic disease, hormonal changes, and medications contribute to anemia (8, 10). The exact mechanisms of anemia in patient with neuropathy and retinopathy is not clear, but anemia may be a sign of longer DM. In other words, longer hyperglycemia in patients with anemia is associated with more frequent microvascular complications. Obesity, insulin resistance, and poor glycemic control lead into inflammatory reactions (24). On the other hand, inflammation decreases erythropoietin gene expression and reduce erythropoietin sensitivity in target cells because of iron deposition in reticuloendothelial system (30). Hyperglycemia raises sorbitol level in RBCs. This impairs Na^+^/K^+^-ATPase activity and consequently leads into osmotic imbalance and cell death (27). As we performed a cross-sectional study, cause-and-effect assessment between microvascular complications and anemia was not possible; however, anemia can affect any organ by inducing hypoxia. Microvascular complication can cause anemia too.

In our study, duration of DM and use of medications like insulin, angiotensin converting enzyme inhibitor and angiotensin receptor blockers were more prevalent in patients with anemia. This can be due to prolonged hyperglycemia that can induce anemia (9, 21, 29). Whatever the mechanism and cause of anemia is, it lowers quality of life and significantly contributes to morbidity and symptoms such as lack of energy and breathlessness in patients with renal impairment (9). Although ADA has not recommended annual screening for presence of anemia (31), with respect to the high prevalence of anemia in patients with DM and its role in morbidity, individualized evaluations for the presence of anemia should be considered. Our study population was large enough to detect clinically important differences; the protocols were easy to follow and we performed the study in a community setting with patients of various socioeconomic classes. Participants’ compliance was high and our research staffs were highly trained. In our study, many outcomes were evaluated. Statistical analyses were straightforward and missing value analysis was not required. We used a single urinary assessment of albuminuria as the basis for diagnosis of nephropathy. Therefore, prevalence of nephropathy may be overestimated in our sample. The cross-sectional design in which one cannot assess cause and effect relationship was another limitation to this study. Anemia is a common finding in patients with DM and could be detected in patients with normal albuminuria and normal renal function; however, albuminuria and decline in GFR are detected frequently in patients with anemia.
